# Dietary Inflammatory Index during Pregnancy and Congenital Heart Defects

**DOI:** 10.3390/nu15102262

**Published:** 2023-05-10

**Authors:** Jiaomei Yang, Qianqian Chang, Qiancheng Du, Shaonong Dang, Lingxia Zeng, Hong Yan

**Affiliations:** 1 Department of Epidemiology and Health Statistics, School of Public Health, Xi’an Jiaotong University Health Science Center, Xi’an 710061, China; cqq20160820@stu.xjtu.edu.cn (Q.C.); duqiancheng@stu.xjtu.edu.cn (Q.D.); tjdshn@mail.xjtu.edu.cn (S.D.); tjzlx@xjtu.edu.cn (L.Z.); xjtu_yh2014@163.com (H.Y.); 2 Nutrition and Food Safety Engineering Research Center of Shaanxi Province, Xi’an 710061, China; 3 Key Laboratory of Environment and Genes Related to Diseases, Xi’an Jiaotong University, Ministry of Education, Xi’an 710061, China

**Keywords:** dietary inflammatory index, congenital heart defects, pregnancy, Chinese

## Abstract

The relationship between diet-related inflammation during pregnancy and congenital heart defects (CHD) is unclear. This study attempted to investigate the association between the dietary inflammation index (DII) during pregnancy, reflecting the overall inflammatory potential of the maternal diet, and CHD in Northwest China. A case-control study with 474 cases and 948 controls was performed in Xi’an City, China. Eligible women awaiting delivery were recruited, and their dietary and other information during pregnancy was collected. Logistic regression models were applied to estimate the risk of CHD in association with DII. The maternal DII ranged from −1.36 to 5.73 in cases, and 0.43 to 5.63 in controls. Pregnant women with per 1 higher DII score were at 31% higher risk of fetal CHD (OR = 1.31, 95%CI = 1.14–1.51), and the adjusted OR (95%CI) comparing the pro-inflammatory diet group with the anti-inflammatory diet group was 2.04 (1.42–2.92). The inverse association of maternal DII score with CHD risk was consistent across various subgroups of maternal characteristics. Maternal DII in pregnancy had good predictive value for CHD in offspring, with the areas under the receiver operating characteristic curve higher than 0.7. These findings suggested that avoiding a pro-inflammatory diet in pregnancy should be emphasized in the prevention of CHD.

## 1. Introduction

Congenital heart defects (CHD) are the most common congenital disorders globally, with the birth prevalence being 9.41‰ worldwide [1] and 9.00‰ in China [2]. It is estimated that millions of neonates are diagnosed with CHD every year worldwide [1], including 0.15 million in China [2]. CHD is the leading cause of infant morbidity and mortality from birth defects, and responsible for more than 0.26 million deaths globally [3], imposing great burdens on the family and society. The etiology for CHD is largely unknown, but previous research has shown that both genetic and environmental factors may contribute to CHD [4]. The major modifiable risk factors for CHD are generally accepted as maternal smoking, alcohol intake, dietary habits, and environmental exposures [4].

Previous studies have reported that maternal intakes of some nutrients, including folic acid, iron, selenium, zinc, and niacin, are associated with fetal CHD [5,6,7,8]. Maternal obesity, diabetes mellitus, and infection during pregnancy are reported to be associated with fetal cardiovascular development [9,10]. These maternal risk factors for CHD are associated with localized and systemic inflammatory cytokine milieu in the placenta and plasma [11]. One study has shown that whole blood cultures derived from mothers with CHD fetuses had higher levels of pro-inflammatory cytokines when activated with mitogen [11], emphasizing the importance of maternal inflammatory conditions in fetal cardiovascular development.

Pregnant women are usually in a low-grade systemic inflammation state due to physiological responses [12]. Diet plays a central role in the regulation of systemic inflammation through pro-inflammatory or anti-inflammatory components of foods and nutrients [13], and is also an important modifiable factor for the prevention of CHD [7,8,14,15,16]. Thus, it is important to investigate the association between pro-inflammatory diet in pregnancy and CHD to provide optimal recommendations for pregnant women to prevent fetal CHD. The Dietary Inflammatory Index (DII) is a literature-derived score for evaluating the overall inflammatory potential of a person’s diet [13]. The DII was determined by peer-reviewed articles about the effect of diet on inflammatory biomarkers [13]. A higher DII score indicates that the diet is pro-inflammatory, while a lower DII score indicates that the diet is anti-inflammatory. The DII has been proven to be of value for the associations with health status in the general population [13], and has also been increasingly used as a predictor of pregnancy outcomes among pregnant women [17,18]. However, to our knowledge there has been no study assessing the association between DII during pregnancy and CHD risk. Previous studies have evaluated some maternal predictors in pregnancy for CHD [14,19,20], giving references for the early prediction of CHD. However, the predictive value of DII for CHD has not been assessed.

The present case-control study in Northwest China attempted to investigate the relationship between DII in pregnancy and CHD and assess the prediction value for DII on CHD.

## 2. Materials and Methods

### 2.1. Study Design and Participants

Between August 2014 and August 2016, we undertook a case-control study in six comprehensive hospitals in Xi’an City, Northwest China. These six hospitals have incorporated fetal echocardiography at 20th–24th gestational weeks into the routine prenatal ultrasound program to screen for CHD. The detailed study design has been reported previously [8,15,16]. Briefly, among pregnant women awaiting delivery in hospitals, those having fetuses with isolated CHD and no genetic malformation were included in the case group, and those having normal fetuses without any birth defects were included in the control group. Pregnant women with multiple pregnancies or diabetes were excluded because of potentially distinct etiologies. Qualified specialists in each hospital strictly enforced the standard criteria to diagnose birth outcomes. We also undertook a follow-up by telephone within one year after birth to confirm the diagnoses. We randomly selected controls in each hospital each month, and the ratio of the number of controls to cases included in the same hospital in the same month was 2:1. To detect a significant (*p* < 0.05) OR of 1.50 between high and low DII score groups with a statistical power of 80%, 305 cases and 610 controls would be required. A total of 474 cases and 948 controls with completed questionnaires were finally included in the analysis, meeting the sample size requirements.

The study was approved by the Xi’an Jiaotong University Health Science Center (No. 2012008). All participants provided informed consent before the survey.

### 2.2. Dietary Assessment and DII Score

We collected maternal diet information throughout pregnancy by face-to-face interviews while awaiting delivery using a semi-quantitative food frequency questionnaire (FFQ). The FFQ consists of 111 food items on the basis of a validated FFQ for pregnant women in Northwest China [21]. Women reported consumption frequency according to eight predefined categories and also recalled the portion sizes with the assistance of food portion images [22,23]. Maternal dietary habits tend to be stable throughout pregnancy [24]; thus, maternal diets throughout pregnancy are comparable with those in the 3rd–8th gestational week, the critical period of fetal cardiovascular development [7,8,15,16]. We applied the Chinese Food Composition Tables to derive maternal nutrient intakes during pregnancy [25,26].

We calculated the DII score using the methods described by Shivappa et al. [13]. We included 30 food parameters to calculate the DII score: 8 pro-inflammatory food parameters (energy, carbohydrate, total fat, protein, cholesterol, saturated fatty acid, vitamin B_12_, and iron) and 22 anti-inflammatory parameters (fiber, monounsaturated fatty acid, polyunsaturated fatty acid, *n*-3 fatty acid, thiamin, riboflavin, vitamin B_6_, folic acid, niacin, β-carotene, vitamin A, vitamin C, vitamin E, zinc, selenium, magnesium, caffeine, alcohol, garlic, onion, green/black tea, and pepper) that were available in the current study. We obtained the z-score by subtracting the “standard global mean” from the consumption amount recalled by each pregnant woman and dividing this value by the standard deviation. To minimize the “right skewness”, this z-score was converted to a centered proportion. We then multiplied this proportion by the respective food parameter effect score according to the study by Shivappa et al. [13]. We finally summed all of the food-parameter-specific DII scores to create the overall DII score for each pregnant woman. In addition, we constructed a Mediterranean Diet Score (MDS) and a Global Diet Quality Score (GDQS) using the FFQ data according to the methods previously reported [14,27,28].

### 2.3. Covariates

Using a structured questionnaire, trained investigators collected the following covariates: (1) sociodemographic characteristics: maternal age, residence, education, work, and parity; (2) maternal health-related factors in early pregnancy: passive smoking, anemia, medication use, and iron/folate supplements use. Maternal age was grouped as two categories (<30 years/≥30 years). Residence included rural and urban areas. Maternal education was divided into two categories (junior high school or below/senior high school or above). Women with no paid employment outside their homes were classified as without employment, otherwise they were classified as in employment. Parity was categorized as two groups (0/≥1). The other covariates were treated as dichotomized factors (no/yes). Women with hemoglobin concentration <110 g/L in pregnancy were diagnosed with anemia.

### 2.4. Statistical Analysis

In univariate comparisons, the χ^2^ test was adopted for categorical variable, and for continuous variables the Kruskal–Wallis test or Mann–Whitney U test was applied because of the non-normal distributions observed by the Shapiro–Wilk test. Considering the clustering in the design through hospitals, mixed logistic regression models were applied to evaluate ORs (95%CIs) for total CHD and CHD subtypes in association with maternal DII during pregnancy. The DII score was divided into three groups according to the 25th percentile and 75th percentile of the control distribution. The anti-inflammatory diet group was defined if the DII score was lower than the 25th percentile, the pro-inflammatory diet group was defined if the DII score was higher than the 75th percentile, and the intermediate group was defined if the DII score was in the range of the 25th percentile and 75th percentile. Potential confounders were controlled in the models if they were important priori confounders [4,8,29] and changed the estimates by more than 10% [30]. P for trend was calculated by including group specific median value in the model. Subgroup analyses were conducted according to maternal characteristics (maternal age, residence, education, occupation, parity, and maternal passive smoking, anemia, medication use, and iron/folate supplement use in early pregnancy). The interaction between maternal DII and each subgroup factors was tested by the likelihood ratio test comparing regression models with and without an interaction term. Sensitivity analyses were also conducted by dividing participants as three groups according to the tertiles of DII score in the control.

The receiver operating characteristic (ROC) curves were established to estimate the optimal cut-off values of DII during pregnancy for total CHD and CHD subtypes with the maximum Youden index. The areas under the ROC curves (AUCs) showed the accuracy of DII as a predictor for CHD. The AUC values indicated the predictive power as follows: >0.9, very good; >0.8, good; and >0.7, useful [31].

All analyses were conducted using the Stata software (version 15.0; StataCorp, College Station, TX, USA). Two-sided statistical significance was set at 0.05.

## 3. Results

### 3.1. Characteristics of the Study Participants

**Figure 1:**
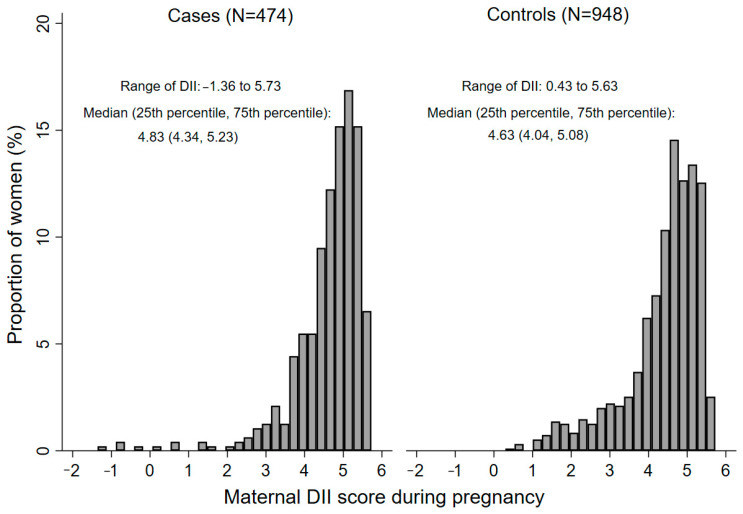
The distribution of DII scores during pregnancy among cases and controls. A significant difference in maternal DII was found between cases and controls by Mann–Whitney U test (*p* < 0.001). DII, Dietary Inflammatory Index.

The distribution of DII scores in pregnancy among cases and controls is shown in Figure 1. The maternal DII ranged from −1.36 to 5.73 in cases, and 0.43 to 5.63 in controls. Pregnant women in the cases had a higher DII score than the controls (*p* < 0.001), with the medians (25th percentile, 75th percentile) being 4.83 (4.34, 5.23) and 4.63 (4.04, 5.08), respectively. The baseline characteristics of the three groups of maternal DII scores are displayed in Table 1. Among the cases, no difference in maternal characteristics existed among the three DII groups. Among the controls, participants in the intermediate group were more likely to be multipara, and mothers with higher DII score were more likely to take iron/folate supplements in early pregnancy. Maternal residence, education, occupation, parity, and maternal passive smoking, anemia, medication use, and iron/folate supplements use in early pregnancy were significantly different between cases and controls (all *p* < 0.05) (Table S1).

### 3.2. Dietary Intakes and Dietary Quality Scores during Pregnancy among the DII Groups

Pregnant women with higher DII score in pregnancy showed lower intakes of main food groups, including grains and tubers, vegetables, fruits, dairy, legumes, meats, fish, eggs, and nuts, both in cases and controls (all *p* < 0.001) (Table 2). Pregnant women with higher DII scores also showed lower MDS and GDQS scores in the case and control groups (all *p* < 0.001) (Table 2). Compared with the controls, case mothers had higher intakes of grains and tubers but lower intakes of other main food groups (all *p* < 0.001), and also had lower MDS and GDQS scores (both *p* < 0.001) (Table S2). Participants with higher DII score during pregnancy reported lower intakes of energy, carbohydrate, total fat, protein, cholesterol, fiber, saturated fatty acid, monounsaturated fatty acid, polyunsaturated fatty acid, *n*-3 fatty acid, vitamins (thiamin (vitamin B_1_), riboflavin (vitamin B_2_), niacin (vitamin B_3_), vitamin B_6_, folic acid (vitamin B_9_), vitamin B_12_, β-carotene, vitamin A, vitamin C, and vitamin E), minerals (iron, zinc, selenium, and magnesium), garlic, onion, and pepper both in cases and controls (Table S3). Participants in the cases consumed lower intakes than the controls of all dietary components included in the DII calculation except carbohydrate, caffeine, alcohol, green/black tea, and pepper (Table S4).

### 3.3. Association between Maternal DII during Pregnancy and CHD

The associations of maternal DII in pregnancy with the risks of total CHD, ventricular septal defects (VSD), and atrial septal defects (ASD) are displayed in Table 3. Compared with those in the anti-inflammatory diet group, mothers in the pro-inflammatory diet group had a higher risk of delivering fetuses with total CHD (OR = 2.04, 95%CI = 1.42–2.92), VSD (OR = 2.00, 95%CI = 1.25–3.19), and ASD (OR = 1.92, 95%CI = 1.22–3.03), with the tests for trend statistically significant (all *p* < 0.05). The risks of total CHD, VSD, and ASD were increased by 31% (OR = 1.31, 95%CI = 1.14–1.51), 29% (OR = 1.29, 95%CI = 1.07–1.55), and 25% (OR = 1.25, 95%CI = 1.04–1.50) for per 1 higher score of maternal DII in pregnancy, respectively.

Subgroup analyses showed that the risks of total CHD, VSD, and ASD in association with maternal DII during pregnancy did not alter by maternal characteristics including maternal age, residence, education, occupation, parity, and maternal passive smoking, anemia, medication use, and iron/folate supplement use in early pregnancy (Figures S1–S3). When dividing participants as three groups according to the tertiles of DII score in the control, compared with the lowest tertile group, the highest tertile group showed higher risks of total CHD (OR = 1.66, 95%CI = 1.22–2.28), VSD (OR = 1.55, 95%CI = 1.03–2.33), and ASD (OR = 1.48, 95%CI = 1.08–2.02), with the tests for trend significant (all *p* < 0.05) (Table S5).

### 3.4. The Prediction Value for Maternal DII during Pregnancy on CHD

**Figure 2:**
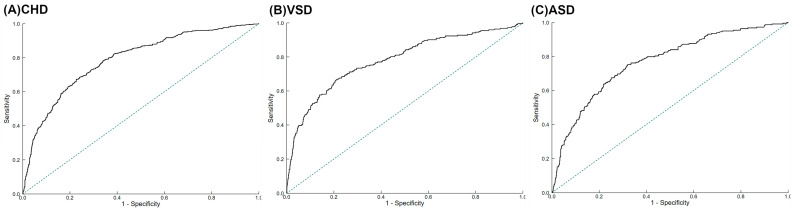
The ROC for Dietary Inflammatory Index in pregnancy in the prediction of (**A**) total congenital heart defects, (**B**) ventricular septal defects, and (**C**) atrial septal defects. ASD, atrial septal defects; CHD, congenital heart defects; ROC, receiver operating characteristic curves; VSD, ventricular septal defects. The dotted line refers to the reference line that results from random selection.

The ROC for maternal DII in pregnancy in the prediction of total CHD, VSD, and ASD is shown in Figure 2. The ROC indicated that maternal DII in pregnancy were useful in predicting total CHD, VSD, and ASD, with the AUC to be 0.79 (0.76, 0.81), 0.78 (0.74, 0.82), and 0.77 (0.73, 0.80), respectively. The optimal DII cut-off values were 5.41 for total CHD (sensitivity: 67.3%, specificity: 77.3%), 5.31 for VSD (sensitivity: 66.7%, specificity: 79.0%), and 5.53 for ASD (sensitivity: 75.2%, specificity: 67.9%), respectively.

## 4. Discussion

In the current case-control study, we found that higher maternal DII scores, indicating a more pro-inflammatory diet, were associated with higher risks of total CHD and its subtypes in fetuses. These inverse associations of DII score in pregnancy with CHD were consistent across various subgroups of maternal characteristics. We also observed that maternal DII in pregnancy had good predictive value for total CHD and its subtypes. To our knowledge, this is the first study to report data on maternal DII in pregnancy and CHD.

Although there has been no study exploring the relationship between maternal DII in pregnancy and CHD, previous research has shown that maternal pro-inflammatory diet in pregnancy is associated with adverse birth outcomes, such as premature birth, low birth weight, and small for gestational age [17,32,33], which are closely related with birth defects. Moreover, several previous studies have reported CHD risk in association with dietary patterns and dietary quality indices during pregnancy [15,34,35], which share some similar dietary components as the DII. For example, the one-carbon-rich dietary pattern during pregnancy, which was high in fish and seafood, was observed to be associated with a lower risk of CHD [35], and the Mediterranean diet during pregnancy, which was high in whole grains, fruits, vegetables, legumes, nuts, and fish, and, high in olive oil but low in saturated lipids, low to moderate in dairy, and limited in red meat, was reported to reduce CHD risk [14,34]. These similar dietary components may explain why those dietary patterns and scores all showed potential health benefits for fetal cardiovascular development. Compared with other dietary scoring systems such as MDS and GDQS that were also reported to show good predictive value for CHD [14], the maternal DII score reflects the inflammation potential of one diet as a whole and has been shown in high relation with maternal cytokine levels such as TNF-α, IL-1β, IL-8, IL-6, IL-10, MCP-1, and C-reactive protein [36,37]. The DII is based on an extensive literature search on the effect of diet on inflammation and is independent on specific means or recommendations of food/nutrient intake [13], which is different from the MDS and GDQS. Considering the importance of maternal inflammatory conditions on fetal cardiovascular development, the DII provides an easy and noninvasive way to assess the dietary inflammatory potential as a predictor for CHD. Findings from the present study imply that it is important to incorporate the suggestion of avoiding a pro-inflammatory diet in routine pregnancy management practices to prevent fetal CHD.

Several mechanisms may explain the higher risk of fetal CHD associated with higher maternal DII during pregnancy. First, the deleterious effect of a pro-inflammatory diet in pregnancy on fetal CHD may come from the increased pro-inflammatory cytokines. One recent study reported that placental inflammatory monocytes of maternal origin could change the cardiac tissue structure by migrating the embryonic heart [38]. Second, the higher systemic inflammation due to higher DII may cause a stress response, further influencing the normal development of the fetal cardiovascular system [39]. Third, it is possible that dietary inflammatory potential during pregnancy participates in the regulation of gut microbiota [40], which was reported to influence fetal CHD [41]. Fourth, the observed relationship between DII and CHD may be partly due to the low dietary quality of a pro-inflammatory diet. Previous research has reported that a higher maternal MDS, indicating a higher dietary quality, was associated with a lower DII score [32] and lower risk of CHD [14,34]. In fact, the present study also showed lower MDS and GDQS scores in the pro-inflammatory diet group and in the case group.

Our study provides valuable evidence on the risk of CHD in association with maternal DII score during pregnancy. However, some limitations merit discussion. First, we cannot exclude recall bias because data in pregnancy was recalled by participants awaiting delivery, although previous research indicated that mothers could recall information in pregnancy well after years [42,43]. Second, we cannot exclude exposure misclassification because we gathered dietary data in the entire pregnancy rather than in the 3rd–8th gestational week, the critical period of fetal cardiovascular development. However, previous research has shown maternal dietary habits are usually stable throughout pregnancy [24]. Third, we cannot exclude selection bias because we did not include CHD fetuses who had died before delivery at term. Fourth, we cannot separately assess the relationships between DII and other CHD subtypes because of the limited sample size. Finally, we cannot rule out the possibility of residual confounders, and cannot uncover a real causal relationship because of the case-control design.

## 5. Conclusions

The present study suggested that a higher DII score during pregnancy, indicating a more pro-inflammatory diet, was associated with higher CHD risk. Furthermore, the maternal DII score in pregnancy had good predictive value for fetal CHD. Our results implied that avoiding a pro-inflammatory diet could be an interesting target for prevention strategies to reduce the incidence of CHD in Northwest China. Routine pregnancy management should emphasize the importance of reducing dietary inflammation to prevent fetal CHD. Further studies are warranted to investigate the validity of the DII as a predicator for CHD in other populations, and further understand the mechanisms associating dietary inflammation in pregnancy with fetal CHD.

## Figures and Tables

**Table 1:** Characteristics of the study participants according to three groups of maternal DII scores during pregnancy.

	Cases (*N* = 474)				Controls (*N* = 948)			
	Anti-Inflammatory Diet Group ^1^ (*N* = 83)	Intermediate Group ^1^ (*N* = 218)	Pro-Inflammatory Diet Group ^1^ (*N* = 173)	*p* ^2^	Anti-Inflammatory Diet Group ^1^ (*N* = 237)	Intermediate Group ^1^ (*N* = 477)	Pro-Inflammatory Diet Group ^1^ (*N* = 234)	*p* ^2^
DII								
Range	−1.36 to 4.04	4.04 to 5.06	5.08 to 5.73		0.43 to 4.04	4.05 to 5.08	5.08 to 5.63	
Median (25th percentile, 75th percentile)	3.55 (2.88, 3.82)	4.66 (4.42, 4.86)	5.30 (5.20, 5.42)	<0.001	3.16 (2.35, 3.77)	4.63 (4.41, 4.84)	5.30 (5.21, 5.42)	<0.001
Sociodemographic characteristics, n (%)								
Maternal age ≥30 years	24 (28.9)	79 (36.2)	56 (32.4)	0.446	77 (32.5)	170 (35.6)	77 (32.9)	0.631
Rural residence	32 (38.6)	81 (37.2)	48 (27.7)	0.093	58 (24.5)	143 (30.0)	68 (29.1)	0.296
Maternal education, senior high school or above	50 (60.2)	136 (62.4)	93 (53.8)	0.218	195 (82.3)	377 (79.0)	193 (82.5)	0.427
Maternal occupation, in employment	42 (50.6)	112 (51.4)	86 (49.7)	0.948	185 (78.1)	388 (81.3)	174 (74.4)	0.096
Nulliparity	53 (63.9)	127 (58.3)	94 (54.3)	0.347	197 (83.1)	367 (76.9)	197 (84.2)	0.033
Maternal health-related factors in early pregnancy, n (%)								
Passive smoking	22 (26.5)	79 (36.2)	58 (33.5)	0.279	17 (7.2)	49 (10.3)	22 (9.4)	0.404
Anemia	8 (9.6)	39 (17.9)	33 (19.1)	0.146	27 (11.4)	48 (10.1)	28 (12.0)	0.713
Medication use	34 (41.0)	88 (40.4)	75 (43.4)	0.832	86 (36.3)	138 (28.9)	64 (27.4)	0.067
Iron/folate supplements use	59 (71.1)	171 (78.4)	133 (76.9)	0.401	204 (86.1)	423 (88.7)	219 (93.6)	0.027

DII, Dietary Inflammatory Index. ^1^ The anti-inflammatory diet group indicates the DII score lower than the 25th percentile of the control distribution, the pro-inflammatory diet group indicates the DII score higher than the 75th percentile of the control distribution, and the intermediate group indicates the DII score in the range of the 25th percentile and 75th percentile of the control distribution. ^2^ *p* values are from *χ^2^* test for categorical variables and from Kruskal–Wallis test for continuous variables.

**Table 2:** Food groups intake and dietary quality scores during pregnancy according to three groups of maternal DII scores during pregnancy.

	Cases (*N* = 474)				Controls (*N* = 948)			
	Anti-Inflammatory Diet Group ^1^ (*N* = 83)	Intermediate Group ^1^ (*N* = 218)	Pro-Inflammatory Diet Group ^1^ (*N* = 173)	*p* ^2^	Anti-Inflammatory Diet Group^1^ (*N* = 237)	Intermediate Group ^1^ (*N* = 477)	Pro-Inflammatory Diet Group ^1^ (*N* = 234)	*p* ^2^
Food groups intake, median (25th percentile, 75th percentile), g/d								
Grains and tubers	352.0 (259.3, 463.6)	244.2 (204.1, 313.8)	186.1 (142.3, 241.3)	<0.001	335.8 (252.1, 440.5)	204.5 (159.9, 280.4)	133.2 (100.4, 163.0)	<0.001
Vegetables	823.8 (590.4, 1084.3)	365.3 (263.0, 448.0)	178.0 (119.5, 214.7)	<0.001	784.3 (548.6, 1340.6)	373.8 (260.8, 460.0)	178.5 (111.6, 214.7)	<0.001
Fruits	563.8 (347.9, 875.0)	327.4 (216.6, 483.5)	162.7 (107.5, 258.3)	<0.001	668.8 (405.6, 915.0)	343.6 (242.6, 490.7)	179.8 (132.9, 254.8)	<0.001
Dairy	128.6 (28.6, 214.3)	85.7 (14.1, 200.0)	14.3 (0, 85.7)	<0.001	172.3 (128.6, 278.6)	172.0 (85.7, 242.9)	100.0 (42.9, 200.0)	<0.001
Legumes	110.7 (60.7, 189.1)	49.9 (24.0, 94.4)	21.4 (8.8, 35.4)	<0.001	192.9 (106.1, 235.7)	78.6 (44.5, 128.6)	36.7 (25.0, 47.9)	<0.001
Meats	78.1 (35.2, 128.6)	38.0 (16.3, 78.6)	20.0 (10.0, 41.0)	<0.001	96.2 (49.1, 156.9)	57.1 (33.3, 92.1)	28.1 (22.1, 41.9)	<0.001
Fish	14.3 (4.0, 31.6)	6.7 (1.3, 17.1)	3.3 (0, 8.0)	<0.001	41.8 (18.3, 85.7)	17.3 (10.1, 33.7)	11.1 (6.7, 16.9)	<0.001
Eggs	25.7 (8.6, 50.0)	21.4 (4.3, 50.0)	21.4 (3.3, 39.3)	<0.001	39.3 (21.4, 50.0)	32.9 (21.4, 50.0)	22.4 (8.5, 50.0)	<0.001
Nuts	18.9 (8.1, 45.0)	12.6 (4.6, 34.0)	3.0 (1.3, 6.4)	<0.001	38.6 (14.1, 71.1)	12.9 (5.5, 33.8)	4.8 (3.3, 8.5)	<0.001
Dietary quality scores, median (25th percentile, 75th percentile)								
MDS	6.0 (5.0, 7.0)	4.0 (3.0, 5.0)	2.0 (1.0, 3.0)	<0.001	7.0 (6.0, 7.0)	5.0 (4.0, 6.0)	2.0 (2.0, 3.0)	<0.001
GDQS	32.8 (29.5, 35.0)	29.3 (26.5, 31.8)	22.5 (20.5, 25.0)	<0.001	34.8 (32.8, 36.9)	31.5 (29.3, 33.5)	25.0 (23.0, 27.0)	<0.001

DII, Dietary Inflammatory Index; MDS, Mediterranean Diet Score; GDQS, Global Diet Quality Score. ^1^ The anti-inflammatory diet group indicates the DII score lower than the 25th percentile of the control distribution, the pro-inflammatory diet group indicates the DII score higher than the 75th percentile of the control distribution, and the intermediate group indicates the DII score in the range of the 25th percentile and 75th percentile of the control distribution. ^2^ *p* values are from Kruskal–Wallis test for continuous variables.

**Table 3:** Associations between DII score during pregnancy and congenital heart defects.

	Anti-Inflammatory Diet Group ^1^	Intermediate Group ^1^	Pro-Inflammatory Diet Group ^1^	*p* for Trend	Per 1 Higher Score
Total congenital heart defects					
*N*_cases_/*N*_controls_	83/237	218/477	173/234	474/948	474/948
Unadjusted OR (95%CI)	1	1.30 (0.97, 1.76)	2.11 (1.54, 2.90)	<0.001	1.32 (1.16, 1.50)
Adjusted OR (95%CI) ^2^	1	1.25 (0.89, 1.74)	2.04 (1.42, 2.92)	<0.001	1.31 (1.14, 1.51)
Ventricular septal defects					
*N*_cases_/*N*_controls_	39/237	100/477	83/234	222/948	222/948
Unadjusted OR (95%CI)	1	1.26 (0.84, 1.90)	2.10 (1.37, 3.21)	0.001	1.30 (1.09, 1.55)
Adjusted OR (95%CI) ^2^	1	1.17 (0.75, 1.81)	2.00 (1.25, 3.19)	0.007	1.29 (1.07, 1.55)
Atrial septal defects					
*N*_cases_/*N*_controls_	42/237	100/477	76/234	218/948	218/948
Unadjusted OR (95%CI)	1	1.18 (0.80, 1.75)	1.83 (1.21, 2.78)	0.009	1.24 (1.05, 1.47)
Adjusted OR (95%CI) ^2^	1	1.13 (0.74, 1.73)	1.92 (1.22, 3.03)	0.011	1.25 (1.04, 1.50)

DII, Dietary Inflammatory Index. ^1^ The anti-inflammatory diet group indicates the DII score lower than the 25th percentile of the control distribution, the pro-inflammatory diet group indicates the DII score higher than the 75th percentile of the control distribution, and the intermediate group indicates the DII score in the range of the 25th percentile and 75th percentile of the control distribution. ^2^ Adjusted for total energy intake, sociodemographic characteristics (maternal age, residence, education, occupation, and parity), and maternal health-related factors in early pregnancy (passive smoking, anemia, medication use, and iron/folate supplements use).

## Data Availability

The datasets in the current study are available from the corresponding author on reasonable request.

## Supplementary Materials

The following supporting information can be downloaded at: https://www.mdpi.com/article/10.3390/nu15102262/s1, Table S1: Characteristics of the study population among cases and controls; Table S2: Food groups intake and dietary quality scores during pregnancy among cases and controls; Table S3: Daily dietary components intake according to three groups of maternal DII during pregnancy; Table S4: Daily dietary components intake during pregnancy among cases and controls; Table S5: Associations between tertiles of maternal DII score during pregnancy and congenital heart defects; Figure S1: Subgroup analyses for the association between per 1 higher score of Dietary Inflammatory Index in pregnancy and the risk of total congenital heart defects; Figure S2: Subgroup analyses for the association between per 1 higher score of Dietary Inflammatory Index in pregnancy and the risk of ventricular heart defects; Figure S3: Subgroup analyses for the association between per 1 higher score of Dietary Inflammatory Index in pregnancy and the risk of atrial heart defects.
