# Supplementary material for: Dietary Inflammatory Index during Pregnancy and Congenital Heart Defects
Source: Nutrients. 2023 May 10;15(10):2262. doi: 10.3390/nu15102262 (PMC10222117; doi:10.3390/nu15102262)
Supplement: Supplementary file 1 [file nutrients-15-02262-s001.zip › nutrients-2367547-supplementary.pdf]

Table S1. Characteristics of the study population among cases and controls.

	Cases ($N = 474$)	Controls ($N = 948$)	P[1]
Sociodemographic characteristics, n (%)			
Maternal age≥30 years	159 (33.5)	324 (34.2)	0.812
Rural residence	161 (34.0)	269 (28.4)	0.030
Maternal education, senior high school or above	279 (58.9)	765 (80.7)	<0.001
Maternal occupation, in employment	240 (50.6)	747 (78.8)	<0.001
Nullparity	274 (57.8)	761 (80.3)	<0.001
Maternal health-related factors in early pregnancy, n (%)			
Passive smoking	159 (33.5)	88 (9.3)	<0.001
Anemia	80 (16.9)	103 (10.9)	0.001
Medication use	197 (41.6)	288 (30.4)	<0.001
Iron/folate supplements use	363 (76.6)	846 (89.2)	<0.001

DII, Dietary Inflammatory Index.

[1] P values are from χ^2 test for categorical variables and from Mann–Whitney U test for continuous variables.

Table S2. Food groups intake and dietary quality scores during pregnancy among cases and controls.

	Cases (*N* = 474)	Controls (*N* = 948)	*P*[1]
Food groups intake, median (25th percentile, 75th percentile), g/d			
Grains and tubers	234.1 (179.0, 314.3)	204.3 (147.3, 305.6)	<0.001
Vegetables	291.1 (189.0, 460.0)	361.4 (214.7, 524.3)	<0.001
Fruits	285.8 (157.1, 453.7)	329.2 (208.0, 574.5)	<0.001
Dairy	42.9 (6.7, 200.0)	130.2 (78.6, 235.7)	<0.001
Legumes	36.7 (18.3, 90.7)	78.6 (36.7, 151.6)	<0.001
Meats	31.8 (14.3, 75.5)	51.7 (28.1, 96.1)	<0.001
Fish	6.7 (0, 15.7)	17.3 (9.7, 37.1)	<0.001
Eggs	21.4 (3.3, 50.0)	39.3 (21.4, 50.0)	<0.001
Nuts	7.3 (2.5, 25.8)	12.3 (4.6, 35.5)	<0.001
Dietary quality scores, median (25th percentile, 75th percentile)			
MDS	4.0 (2.0, 5.0)	5.0 (3.0, 6.0)	<0.001
GDQS	27.5 (23.7, 31.0)	31.0 (27.3, 34.3)	<0.001

MDS, Mediterranean Diet Score; GDQS, Global Diet Quality Score.

[1] *P* values are from Mann–Whitney U test for continuous variables.

Table S3. Daily dietary components intake according to three groups of maternal DII during pregnancy[1].

	Cases ($N = 474$)				Controls ($N = 948$)			
	Anti-inflammatory diet group[2] ($N = 83$)	Intermediate group[2] ($N = 218$)	Pro-inflammatory diet group[2] ($N = 173$)	P^3	Anti-inflammatory diet group[2] ($N = 237$)	Intermediate group[2] ($N = 477$)	Pro-inflammatory diet group[2] ($N = 234$)	P^3
**Pro-inflammatory food parameters**								
Energy, kcal	2415.9 (2067.6, 2932.6)	1862.7 (1663.8, 2074.8)	1400.1 (1244.7, 1567.3)	<0.001	2740.0 (2379.2, 3224.6)	1909.6 (1709.9, 2237.2)	1379.9 (1235.5, 1517.3)	<0.001
Carbohydrate, g	294.6 (243.0, 373.4)	201.5 (169.5, 235.5)	136.4 (112.5, 165.1)	<0.001	324.9 (262.5, 394.8)	193.4 (163.3, 239.5)	119.0 (98.2, 137.5)	<0.001
Total fat, g	54.6 (40.2, 71.5)	37.3 (23.6, 48.4)	18.4 (13.2, 26.7)	<0.001	67.9 (51.7, 84.7)	42.1 (33.1, 54.4)	23.7 (18.3, 29.5)	<0.001
Protein, g	79.3 (63.5, 99.9)	48.4 (41.7, 58.9)	29.9 (23.4, 36.5)	<0.001	93.9 (77.9, 126.0)	57.2 (47.5, 68.9)	33.2 (26.8, 38.3)	<0.001
Cholesterol, mg	256.1 (126.6, 325.8)	185.6 (69.3, 297.9)	113.4 (33.3, 219.6)	<0.001	323.7 (241.7, 403.0)	283.8 (169.7, 327.2)	168.1 (92.5, 282.5)	<0.001
SFA, g	20.5 (16.2, 30.6)	15.0 (9.7, 20.0)	8.4 (5.7, 12.0)	<0.001	27.4 (19.9, 35.6)	17.9 (14.2, 23.5)	11.1 (8.7, 14.6)	<0.001
Vitamin B_{12}, μg	0.2 (0, 0.4)	0.1 (0, 0.2)	0 (0, 0.1)	<0.001	0.3 (0.1, 0.9)	0.2 (0.1, 0.4)	0.2 (0.1, 0.3)	<0.001
Iron, mg	29.3 (24.1, 39.2)	19.0 (14.5, 22.5)	12.7 (8.1, 16.4)	<0.001	35.1 (28.3, 44.9)	20.5 (17.0, 25.3)	11.2 (8.7, 14.8)	<0.001
**Anti-inflammatory food parameters**								
Fiber, g	36.3 (30.7, 45.5)	21.0 (18.3, 24.0)	13.5 (11.5, 15.5)	<0.001	39.7 (31.8, 52.1)	20.8 (17.2, 24.6)	11.5 (9.2, 13.1)	<0.001
MUFA, g	13.5 (8.1, 18.4)	8.2 (5.0, 12.9)	4.0 (2.5, 6.4)	<0.001	15.4 (11.3, 20.3)	9.9 (7.4, 13.6)	5.7 (4.2, 7.2)	<0.001
PUFA, g	12.6 (9.7, 15.5)	7.4 (5.5, 9.8)	3.3 (2.6, 4.3)	<0.001	17.0 (12.9, 20.7)	8.1 (6.2, 10.7)	3.7 (3.2, 4.4)	<0.001
n-3 fatty acids, g	1.6 (1.2, 2.7)	1.0 (0.7, 1.9)	0.4 (0.3, 0.6)	<0.001	2.8 (2.1, 4.0)	1.2 (0.8, 1.9)	0.5 (0.4, 0.7)	<0.001
Thiamin, mg	0.9 (0.7, 1.1)	0.5 (0.4, 0.6)	0.3 (0.3, 0.4)	<0.001	1.0 (0.8, 1.2)	0.6 (0.5, 0.7)	0.3 (0.3, 0.4)	<0.001
Riboflavin, mg	1.2 (0.9, 1.6)	0.6 (0.5, 0.8)	0.3 (0.3, 0.4)	<0.001	1.4 (1.1, 1.7)	0.8 (0.7, 1.0)	0.5 (0.4, 0.6)	<0.001
Vitamin B_6, mg	1.1 (0.9, 1.4)	0.6 (0.5, 0.7)	0.3 (0.3, 0.4)	<0.001	1.2 (0.9, 1.5)	0.6 (0.5, 0.7)	0.3 (0.2, 0.4)	<0.001
Folic acid, μg	217.8 (187.3, 297.7)	106.0 (88.2, 131.8)	56.0 (44.4, 70.2)	<0.001	236.2 (177.3, 316.4)	121.4 (98.1, 146.2)	66.5 (53.2, 79.5)	<0.001
Niacin, mg	17.1 (13.1, 24.5)	10.4 (8.5, 13.0)	7.1 (5.4, 8.7)	<0.001	21.3 (16.7, 27.4)	12.6 (10.4, 15.6)	7.1 (5.5, 8.7)	<0.001
β-Carotene, μg	4808.8 (4046.4, 6635.4)	1967.3 (1364.5, 2468.6)	855.1 (626.9, 1080.2)	<0.001	4371.5 (2823.9, 6709.0)	1844.8 (1266.2, 2315.9)	896.8 (635.1, 1083.2)	<0.001

Vitamin A, μg RE	962.0 (645.7, 1532.5)	379.8 (269.7, 508.5)	198.5 (137.6, 265.1)	<0.001	921.3 (642.2, 1280.9)	422.8 (322.1, 555.5)	253.8 (202.4, 324.2)	<0.001
Vitamin C, mg	165.3 (121.0, 225.5)	74.3 (59.7, 102.9)	37.8 (28.1, 47.4)	<0.001	176.0 (126.8, 263.8)	77.4 (61.2, 103.7)	45.5 (36.4, 52.3)	<0.001
Vitamin E, mg	24.6 (19.1, 32.8)	13.7 (10.8, 18.1)	6.0 (4.6, 7.9)	<0.001	33.7 (26.5, 42.4)	15.6 (12.6, 19.4)	7.4 (6.2, 8.6)	<0.001
Zinc, mg	9.5 (7.5, 11.7)	5.3 (4.3, 6.63)	2.8 (2.2, 3.8)	<0.001	11.3 (9.1, 15.6)	6.4 (5.3, 7.8)	3.6 (3.0, 4.4)	<0.001
Selenium, μg	41.0 (32.1, 50.4)	24.2 (18.9, 32.8)	14.7 (11.1, 19.5)	<0.001	49.4 (37.4, 72.6)	31.3 (24.5, 39.6)	18.5 (14.9, 23.0)	<0.001
Magnesium, mg	435.3 (373.7, 542.0)	235.9 (204.8, 278.4)	131.3 (105.9, 153.0)	<0.001	484.2 (402.7, 667.0)	254.2 (210.4, 304.2)	139.5 (113.4, 158.6)	<0.001
Caffeine, g	0 (0, 0)	0 (0, 0)	0 (0, 0)	0.550	0 (0, 0)	0 (0, 0)	0 (0, 0)	0.513
Alcohol, g	0 (0, 0)	0 (0, 0)	0 (0, 0)	0.690	0 (0, 0)	0 (0, 0)	0 (0, 0)	0.598
Garlic, g	4.3 (0.7, 10.0)	1.4 (0.7, 7.9)	0.7 (0.0, 1.4)	<0.001	4.3 (1.4, 10.0)	1.4 (0.7, 10.0)	0.7 (0.3, 1.4)	<0.001
Onion, g	14.3 (6.7, 42.9)	6.7 (3.3, 14.3)	3.3 (0.0, 6.7)	<0.001	14.3 (6.7, 42.9)	6.9 (3.3, 14.3)	6.7 (3.3, 6.7)	<0.001
Green/black tea, g	0 (0, 0)	0 (0, 0)	0 (0, 0)	0.691	0 (0, 0)	0 (0, 0)	0 (0, 0)	0.973
Pepper, g	10.7 (3.3, 21.4)	3.6 (1.7, 10.7)	1.7 (1.3, 3.3)	<0.001	10.7 (3.3, 21.4)	3.6 (1.7, 7.1)	3.3 (1.7, 3.3)	<0.001

DII, Dietary Inflammatory Index; MUFA, monounsaturated fatty acid; PUFA, polyunsaturated fatty acid; RE, retinol equivalent; SFA, saturated fatty acid.

[1] Variables are present as median (25th percentile, 75th percentile).

[2] The anti-inflammatory diet group indicates the DII score lower than the 25th percentile of the control distribution, the pro-inflammatory diet group indicates the DII score higher than the 75th percentile of the control distribution, and the intermediate group indicates the DII score in the range of the 25th percentile and 75th percentile of the control distribution.

[3] *P* values are from Kruskal-Wallis test for continuous variables.

Table S4. Daily dietary components intake during pregnancy among cases and controls[1].

	Cases (N = 474)	Controls (N = 948)	P^2
**Pro-inflammatory food parameters**			
Energy, kcal	1753.2 (1452.4, 2086.1)	1907.1 (1563.3, 2415.9)	< 0.001
Carbohydrate, g	185.6 (142.0, 237.7)	190.9 (142.5, 269.8)	0.082
Total fat, g	30.9 (19.0, 47.8)	41.7 (29.0, 59.5)	< 0.001
Protein, g	44.5 (32.0, 60.5)	56.9 (40.9, 78.9)	< 0.001
Cholesterol, mg	154.3 (51.0, 285.0)	278.2 (153.0, 329.8)	< 0.001
SFA, g	13.1 (8.1, 19.3)	17.4 (12.6, 25.0)	< 0.001
Vitamin B_{12}, μg	0.1 (0.0, 0.2)	0.2 (0.1, 0.4)	< 0.001
Iron, mg	17.5 (12.6, 23.3)	20.4 (14.3, 28.9)	< 0.001
**Anti-inflammatory food parameters**			
Fiber, g	19.2 (14.4, 25.7)	20.8 (14.2, 28.8)	0.022
MUFA, g	6.9 (3.9, 12.2)	9.8 (6.6, 14.4)	< 0.001
PUFA, g	6.1 (3.8, 9.8)	8.1 (4.9, 12.9)	< 0.001
n-3 fatty acids, g	0.8 (0.4, 1.6)	1.2 (0.7, 2.3)	< 0.001
Thiamin, mg	0.5 (0.3, 0.7)	0.6 (0.4, 0.8)	< 0.001
Riboflavin, mg	0.6 (0.4, 0.8)	0.8 (0.6, 1.1)	< 0.001
Vitamin B_6, mg	0.5 (0.4, 0.8)	0.6 (0.4, 0.9)	< 0.001
Folic acid, μg	95.1 (61.3, 142.9)	120.2 (81.5, 170.5)	< 0.001
Niacin, mg	9.6 (7.3, 13.2)	12.5 (9.0, 17.3)	< 0.001
β-Carotene, μg	1491.7 (938.6, 2754.3)	1777.3 (1081.6, 2666.3)	0.015
Vitamin A, μg RE	340.2 (208.7, 559.0)	431.2 (295.5, 685.2)	< 0.001
Vitamin C, mg	63.5 (42.0, 107.0)	77.0 (51.8, 123.2)	< 0.001
Vitamin E, mg	11.5 (6.9, 18.2)	15.6 (9.5, 23.5)	< 0.001
Zinc, mg	4.7 (3.1, 6.8)	6.4 (4.6, 9.1)	< 0.001
Selenium, μg	22.7 (15.0, 32.5)	30.8 (21.9, 43.7)	< 0.001
Magnesium, mg	213.2 (144.9, 292.1)	252.4 (171.9, 365.4)	< 0.001
Caffeine, g	0 (0, 0)	0 (0, 0)	0.478
Alcohol, g	0 (0, 0)	0 (0, 0)	0.650
Garlic, g	1.0 (0.3, 4.3)	1.4 (0.7, 8.6)	< 0.001
Onion, g	6.7 (3.3, 14.3)	6.7 (3.3, 14.3)	< 0.001
Green/black tea, g	0 (0, 0)	0 (0, 0)	0.224
Pepper, g	3.3 (1.7, 7.1)	3.5 (1.7, 7.1)	0.231

MUFA, monounsaturated fatty acid; PUFA, polyunsaturated fatty acid; RE, retinol equivalent; SFA, saturated fatty acid.

[1] Variables are present as median (25th percentile, 75th percentile).

[2] P values are from Mann–Whitney U test for continuous variables.

Table S5. Associations between tertiles of maternal DII score during pregnancy and congenital heart defects.

	DII			
	Tertile 1	Tertile 2	Tertile 3	*P* for trend
**Total congenital heart defects**				
$N_{cases}/N_{controls}$	114/314	150/318	210/316	474/948
Unadjusted OR (95%CI)	1	1.30 (0.97, 1.73)	1.83 (1.39, 2.41)	<0.001
Adjusted OR (95%CI)[1]	1	1.19 (0.86, 1.65)	1.66 (1.22, 2.28)	0.002
**Ventricular septal defects**				
$N_{cases}/N_{controls}$	54/314	70/318	98/316	222/948
Unadjusted OR (95%CI)	1	1.28 (0.87, 1.89)	1.78 (1.23, 2.57)	0.003
Adjusted OR (95%CI)[1]	1	1.15 (0.75, 1.76)	1.55 (1.03, 2.33)	0.040
**Atrial septal defects**				
$N_{cases}/N_{controls}$	58/314	70/318	90/316	218/948
Unadjusted OR (95%CI)	1	1.19 (0.81, 1.74)	1.54 (1.07, 2.22)	0.022
Adjusted OR (95%CI)[1]	1	1.09 (0.72, 1.64)	1.48 (1.08 2.02)	0.041

DII, Dietary Inflammatory Index.

[1]Adjusted for total energy intake, sociodemographic characteristics (maternal age, residence, education, occupation, and parity), and maternal health-related factors in early pregnancy (passive smoking, anemia, medication use, and iron/folate supplements use).

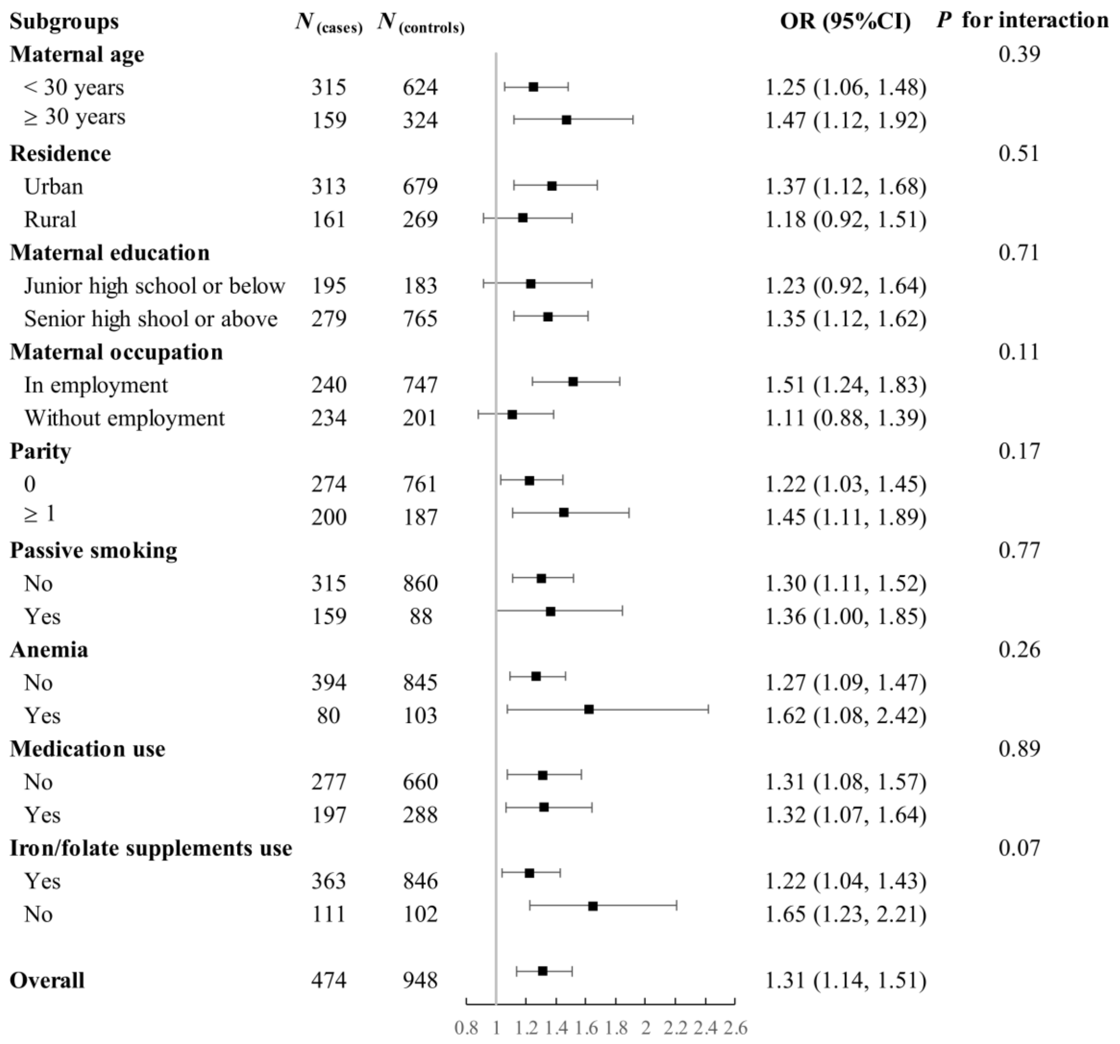

Figure S1. Subgroup analyses for the association between per 1 higher score of Dietary Inflammatory Index in pregnancy and the risk of total congenital heart defects. Analyses were adjusted for total energy intake, sociodemographic characteristics (maternal age, residence, education, occupation, and parity), and maternal health-related factors in early pregnancy (passive smoking, anemia, medication use, and iron/folate supplements use).

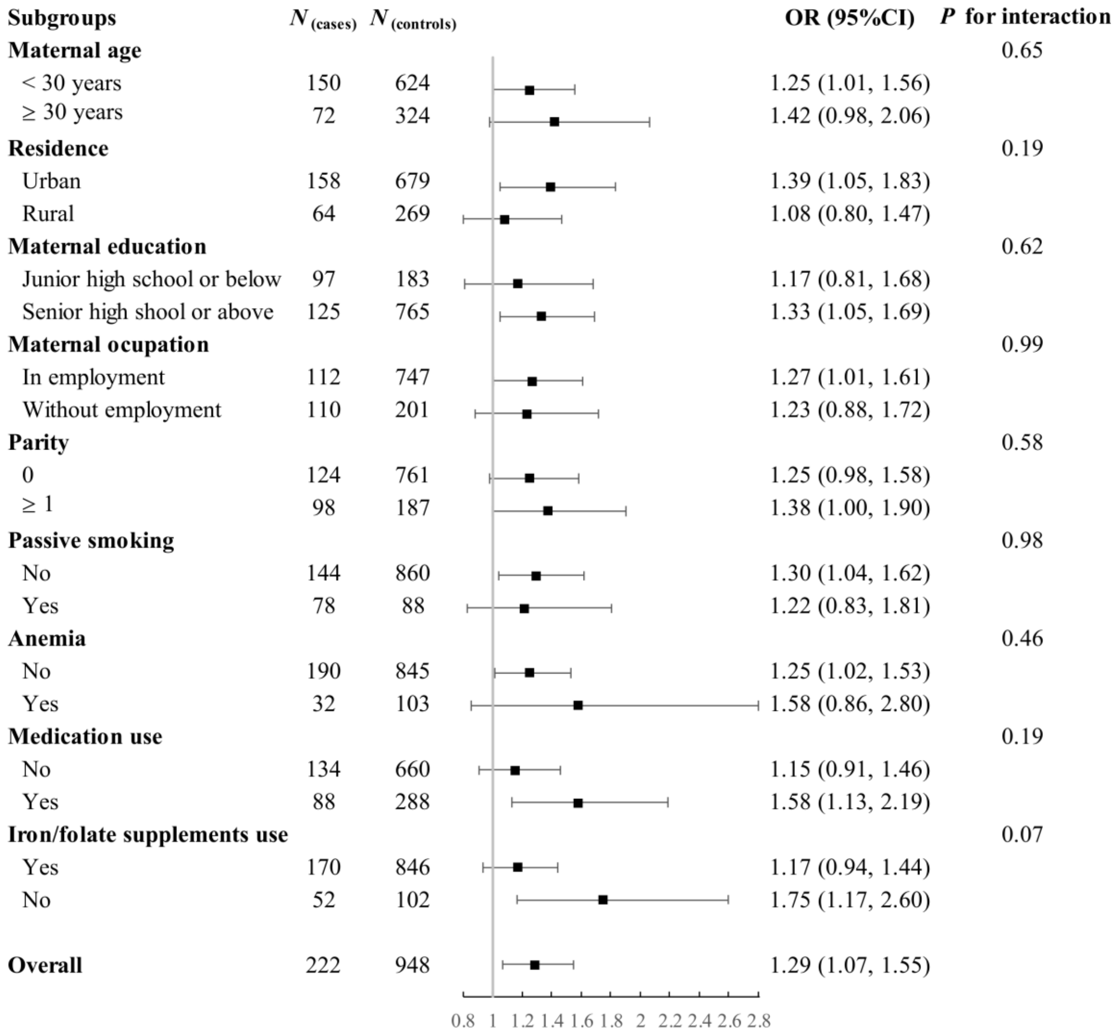

Figure S2. Subgroup analyses for the association between per 1 higher score of Dietary Inflammatory Index in pregnancy and the risk of ventricular heart defects. Analyses were adjusted for total energy intake, sociodemographic characteristics (maternal age, residence, education, occupation, and parity), and maternal health-related factors in early pregnancy (passive smoking, anemia, medication use, and iron/folate supplements use).

Subgroups	$N_{(cases)}$	$N_{(controls)}$	OR (95%CI)	*P* for interaction
**Maternal age**				0.99
< 30 years	145	624	1.25 (1.01, 1.56)	
≥ 30 years	73	324	1.24 (0.89, 1.73)	
**Residence**				0.15
Urban	136	679	1.37 (1.06, 1.79)	
Rural	82	269	1.06 (0.78, 1.45)	
**Maternal education**				0.40
Junior high school or below	70	183	1.09 (0.74, 1.59)	
Senior high shool or above	148	765	1.34 (1.07, 1.70)	
**Maternal occupation**				0.17
In employment	122	747	1.37 (1.07, 1.75)	
Without employment	96	201	0.96 (0.71, 1.30)	
**Parity**				0.17
0	123	761	1.15 (0.93, 1.43)	
≥ 1	95	187	1.40 (0.99, 1.97)	
**Passive smoking**				0.73
No	153	860	1.26 (1.02, 1.56)	
Yes	65	88	1.21 (0.83, 1.77)	
**Anemia**				0.19
No	181	845	1.18 (0.97, 1.44)	
Yes	37	103	1.73 (1.04, 2.80)	
**Medication use**				0.77
No	126	660	1.24 (0.98, 1.58)	
Yes	92	288	1.33 (1.00, 1.77)	
**Iron/folate supplements use**				0.12
Yes	171	846	1.15 (0.93, 1.41)	
No	47	102	1.65 (1.10, 2.48)	
**Overall**	218	948	1.25 (1.04, 1.50)	

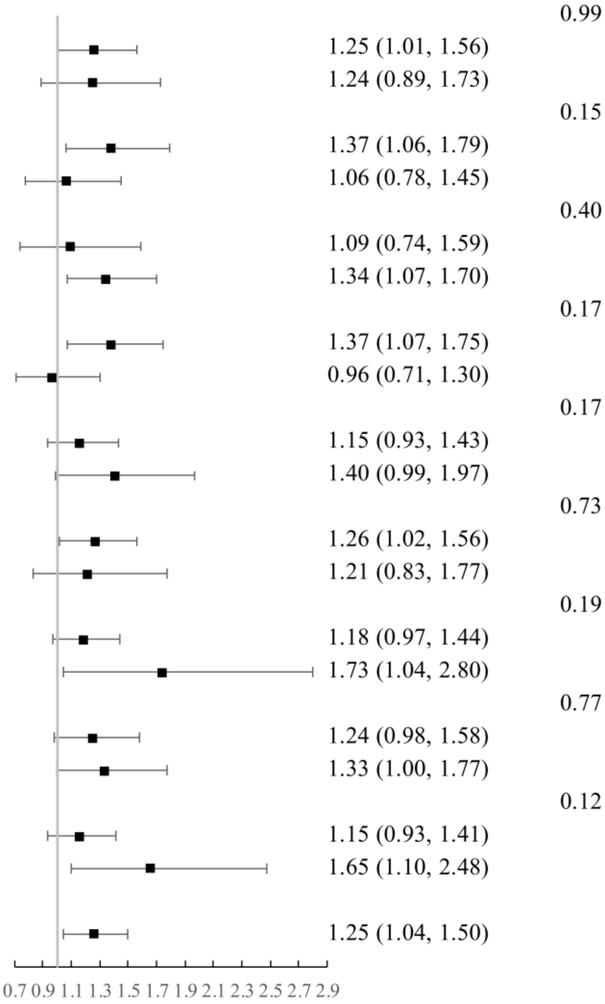

Figure S3. Subgroup analyses for the association between per 1 higher score of Dietary Inflammatory Index in pregnancy and the risk of atrial heart defects. Analyses were adjusted for total energy intake, sociodemographic characteristics (maternal age, residence, education, occupation, and parity), and maternal health-related factors in early pregnancy (passive smoking, anemia, medication use, and iron/folate supplements use)
